# Low Power Near Field Communication Methods for RFID Applications of SIM Cards

**DOI:** 10.3390/s17040867

**Published:** 2017-04-14

**Authors:** Yicheng Chen, Zhaoxia Zheng, Mingyang Gong, Fengqi Yu

**Affiliations:** 1 Shenzhen Institutes of Advanced Technology, Chinese Academy of Sciences, Shenzhen 518055, China; chenyicheng@zkxltech.com; 2 School of Optical and Electronic Information, Huazhong University of Science & Technology, Wuhan 430074, China; zxzheng@hust.edu.cn (Z.Z.); gongmingyang@zkxltech.com (M.G.)

**Keywords:** subscriber identity module (SIM), near field communication, radio frequency identification, low-power design

## Abstract

Power consumption and communication distance have become crucial challenges for SIM card RFID (radio frequency identification) applications. The combination of long distance 2.45 GHz radio frequency (RF) technology and low power 2 kHz near distance communication is a workable scheme. In this paper, an ultra-low frequency 2 kHz near field communication (NFC) method suitable for SIM cards is proposed and verified in silicon. The low frequency transmission model based on electromagnetic induction is discussed. Different transmission modes are introduced and compared, which show that the baseband transmit mode has a better performance. The low-pass filter circuit and programmable gain amplifiers are applied for noise reduction and signal amplitude amplification. Digital-to-analog converters and comparators are used to judge the card approach and departure. A novel differential Manchester decoder is proposed to deal with the internal clock drift in range-controlled communication applications. The chip has been fully implemented in 0.18 µm complementary metal-oxide-semiconductor (CMOS) technology, with a 330 µA work current and a 45 µA idle current. The low frequency chip can be integrated into a radio frequency SIM card for near field RFID applications.

## 1. Introduction

As communication and computing performance evolves, smart phones with various types of sensors have the potential to become identification cards and credit cards. Radio frequency identification (RFID) [1], micro payment [2,3], physical access control [4], location and activity recognition applications [5,6] based on mobile phones are proposed. This is so that the users have no need to keep more cards and keys in their wallet or pocket as the number of services increases. The 13.56 MHz near field communication (NFC) technology based on ISO 14443 protocol is the popular scheme for mobile phones, and this uses the NFC chip, subscriber identity module (SIM) cards, and the single wire protocol (SWP) interface [3,7]. Secure element (SE) chips in the SIM card are used for security assurance, and have usually passed the Common Criteria evaluation assurance level (EAL) 5+ standard and have the same security level as bank smartcards [8].

The 13.56 MHz NFC technology in mobile phones is confronted by two problems in many usage scenarios. Firstly, it is not suitable for middle and long distance RFID applications, such as garage door control and indoor location tracking [9,10,11]. Secondly, the metallic shells of phones can cause a frequency shift for 13.56 MHz technology and a reduction of the field intensity, which can affect the communication quality for NFC devices [12,13]. To solve these problems, 2.45 GHz radio frequency (RF) chips are introduced into the mobile phones; in particular, secure digital (SD) cards and SIM cards are used as substrate for 2.45 GHz chips and antennas [14,15,16]. When a 2.45 GHz RF chip is integrated into the SIM card, there are still two crucial problems for RFID SIM cards: high power consumption and an uncontrollable working distance of up to tens of meters for the 2.45 GHz chip. How to deal with the dilemma between the short distance and long distance applications is a difficult challenge.

This paper presents a strategy to tackle the dilemma, and an improved constitution of RFID SIM cards is proposed. In this type of RFID SIM card, a 2.45 GHz RF chip and an SE chip are integrated. However, the 13.56 MHz technology is not chosen for RFID SIM cards, because the coil of 13.56 MHz is fairly large and is difficult to integrate into SIM cards, especially nano-SIMs. In addition, the metallic shells and card slots of mobile phones can affect the communication quality of 13.56 MHz. Therefore, a low power near-distance communication chip is discussed and customized. Compared to 13.56 MHz NFC chips, the customized low frequency (LF) chip can get energy directly from the mobile phone, so there is no need for the complex amplitude limiter, rectifier, and voltage stabilizing circuits. Besides this, the LF chip is implemented with the function of receiving data only, which results in the removal of transmitting circuits and the reduction of power consumption.

The remainder of this article is organized as follows. Section 2 compares the ordinary SIM and RFID SIM, and describes an improved RFID SIM structure including the low frequency chip. Section 3 briefs the basics of the low frequency data transmission model. The real low frequency communication system design is discussed in Section 4, which describes the frontend circuit structure of the LF chip, the implementation of programmable gain amplifiers, and an improved differential Manchester decoder. The low frequency chip is implemented and tested; the results are shown in Section 5. Finally, concluding remarks are made in Section 6.

## 2. An Improved RFID SIM Structure

Traditional SIM, machine-to-machine (M2M) SIM [17,18], and e-SIM cards [19,20,21,22] perform user identification, M2M communication, and remote provisioning by the SE chip. The SE chip incorporates a secure ARM processor, embedded flash, cryptographic engines, and some interfaces. One of the interfaces is compatible with the ISO/IEC 7816-3 standard for the communication between the mobile phone and the SIM card.

The first generation RFID SIM integrated a secure chip, a 2.45 GHz RF chip, and a 2.45 GHz RF antenna [14,16]. The SE chip configures and controls the 2.45 GHz RF chip via a serial peripheral interface (SPI). It works well in many middle and long distance RFID applications, such as staff attendance. However, for some short distance scenarios, for example, ticket payments for buses and subways, the transaction distance of the micro-payment system must be restricted to about 10 cm. It is a crucial challenge for 2.45 GHz RF communication to make sure that the transactions do not occur beyond a certain range. One scheme is that a near field state indicator can be applied to inform the SE chip and the RF chip.

**Figure 1:**
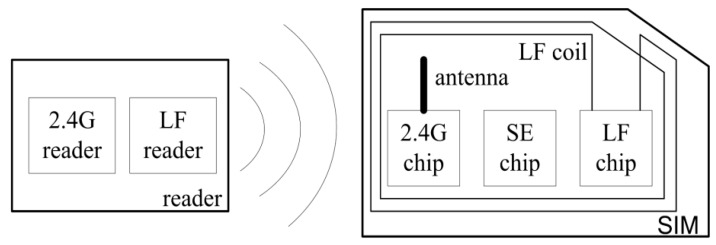
The dual-mode reader and radio frequency identification (RFID) SIM card.

As such, second generation RFID SIM cards and dual-mode readers have been designed, as shown in Figure 1. The dual-mode reader consists of a high frequency reader and a low frequency reader, which can transmit 2.45 GHz and 2 kHz electromagnetic signals. The 2.45 GHz RF chip, the SE chip, the low frequency chip, and the 2.45 GHz and LF antennas constitute the RFID SIM. This type of RFID SIM can be embedded in common SIM, M2M, and e-SIM cards. The SE chip deals with the subscriber identity operations and controls a 2.45 GHz and low frequency chip. The LF chip can only receive data within a short communication distance and with less working current. The 2.45 GHz RF chip can transmit and receive data through different frequency channels and signal strengths.

In middle and long distance RFID applications, the 2.45 GHz RF chip is woken periodically and is sent identification data during its working states. For short distance applications, the typical process is as follows:

(1) First, the 2.45 GHz RF chip sleeps with about 1 µA current, and the LF reader sends 2 kHz signals;
(2) Secondly, the LF chip determines whether or not a card approaches, receives the LF data, and wakes up the 2.45 GHz chip;
(3) Finally, the 2.45 GHz chip requests the reader to suspend the transmission of LF signals and handles 2.45 GHz transactions.

## 3. Low Frequency Data Transmission Model

**Figure 2:**
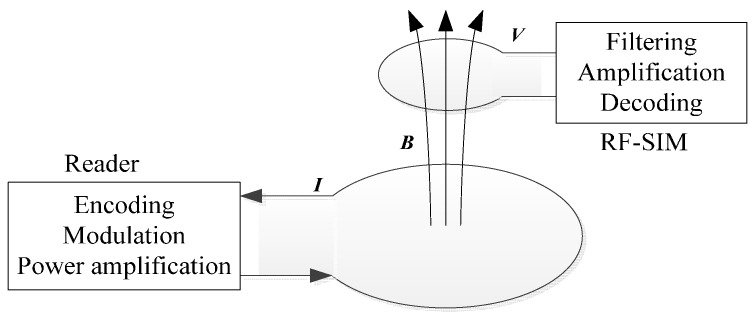
The low frequency data transmission process.

Figure 2 describes the data transmission process between the reader and the RFID SIM card. In the low frequency mode, the raw data is encoded and modulated by the microcontroller unit (MCU) in the reader. The waveforms generated by the MCU are amplified by operational amplifiers, and then transmitted through the antenna. The electromotive force in the coils of the RFID SIM card is generated by electromagnetic induction. Because the operation frequency is distinctly lower and the coils in the SIM cards are much smaller compared to the NFC technology at 13.56 MHz, the electromotive signal is very weak and needs to be handled with caution. Suitable matching and filter circuits between the coils and the LF chip should be designed carefully, as these become an influential factor on the working distance and decoding success ratio. After that, the voltage signal magnification is required, from a few tens of microvolt to several volts. The amplified signals are digitalized by a digital-to-analog converter (DAC) and decoded through a differential Manchester decoder.

For simplicity, considering that the reader antenna is circular, the low frequency magnetic field intensity (*H*) created by the reader is given as [23]

(1) H=INR22(R2+X2)3

where *I* denotes the current of the reader coil, *N* is the number of turns of the reader coil, *R* is the coil radius, and *X* means the distance from the center of the coil along the direction of the *X* axis.

For SIM cards, the magnetic field intensity (*H*) through the LF coil can be assumed to be equal at every point due to the small area and large number of turns of the LF coil. Assuming the SIM LF coil with the area *S* is parallel to the reader coil, the magnetic flux (*Φ*) of the LF coil can be expressed as

(2) Φ=∫B·dS≈μHS

where *B* means magnetic flux density, *μ* denotes magnetic permeability, and · is the inner product. When the number of turns of the LF coil is *M*, the induced voltage (*V*) of the LF coil by magnetic coupling is defined as

(3) V=MdΦdt≈MNμSR22(R2+X2)3dIdt

**Figure 3:**
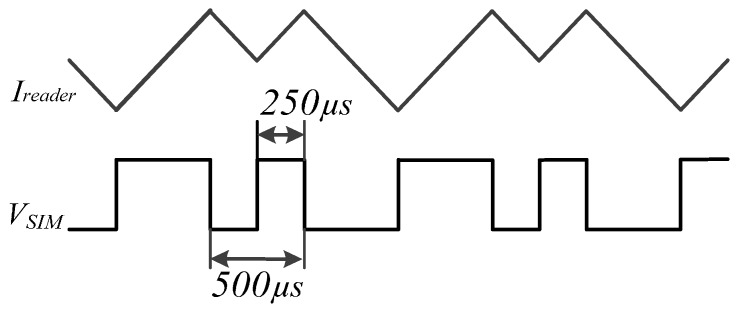
The 2 kHz transmission method for SIM cards by magnetic coupling.

Equation (3) demonstrates that the induced voltage of the LF antenna in the SIM card is proportional to the differential of the reader coil current. Accordingly, if the induced voltage (*V_SIM_*) needs to satisfy a differential Manchester code format, the current (*I_reader_*) in the reader coil needs to be the integral pattern of the corresponding Manchester codes. Each bit of differential Manchester code consumes 500 µs in the SIM card, and the corresponding saw tooth wave of the reader current has the same timing constraint, which is illustrated in Figure 3.

We constructed an experimental prototype by using MCU, operational amplifiers, coils, capacitors, and resistors according to the above low frequency transmission model. The reader current (*I_reader_*) is generated according to Equation (3) and Figure 3. Because magnetic permeability in air is very small, in this method, the induced electromotive signal is very weak, usually a few tens of micro volts. For example, the reader coil is 60 mm by 60 mm with 88 turns, and the coil in the SIM card is 12.3 mm by 8.8 mm with 18 turns. The communication distance is 8 cm between the reader and the SIM card. The voltage peak-to-peak value of the reader is 3.8 V and the impedance of the reader coil is 56 ohm. The induced voltage of the SIM card is about 50 µV according to Equation (3).

The signal cannot obtain enough voltage strength from the electromagnetic induction reader, so the LF chip needs powerful amplifiers to deal with it. LF communication is significantly different from common near field communication such as ISO/IEC 14443 and 15693. Furthermore, the signal strength is influenced by the area and number of turns of the reader coil and the SIM card coil, the working distance, and the angle between the reader coil and the SIM card coil. When the induced signal is magnified significantly and enters a state of saturation, the influence of the above factors can be eliminated within a certain range. This approach needs to design appropriate amplifiers, which is important for the working distance and the decoding stability.

## 4. Low Frequency Communication Implementation

### 4.1. The Frontend Circuit Design

**Figure 4:**
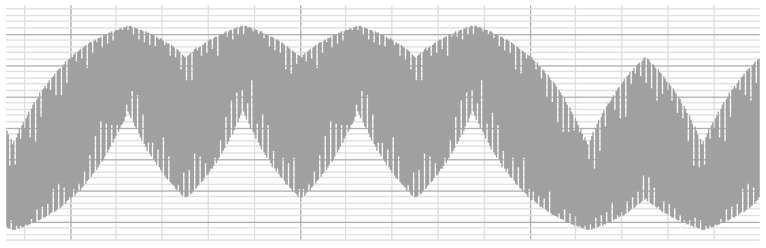
The current wave of the reader coil in pulse density modulation mode.

According to the low frequency data transmission model, the current (*I_reader_*) in the reader coil needs to satisfy the saw tooth wave form. The reader can generate this current wave via the MCU and the operational amplifier. Another method is to modulate the current wave by pulse width or pulse density. The modulated signal is amplified by the operational amplifier, and sent by the reader. Figure 4 shows the current wave of the reader coil in pulse density modulation (PDM) mode. We compare the transmission distances for the same SIM card in three different modes: baseband transmission (BBT), pulse width modulation (PWM), and PDM with a 4 MHz electromagnetic wave as the carrier. Our test results are introduced in Table 1, which shows that the baseband transmission and the PDM mode have about the same transmission distance which is much longer than that of the PWM mode. The micro-SIM has a larger outline dimension and antenna size than the nano-SIM, so the low frequency circuit of the micro-SIM has the furthest working distance. When using other carrier frequencies, such as 2 MHz, 6 MHz, and 8 MHz, similar results are obtained.

**Figure 5:**
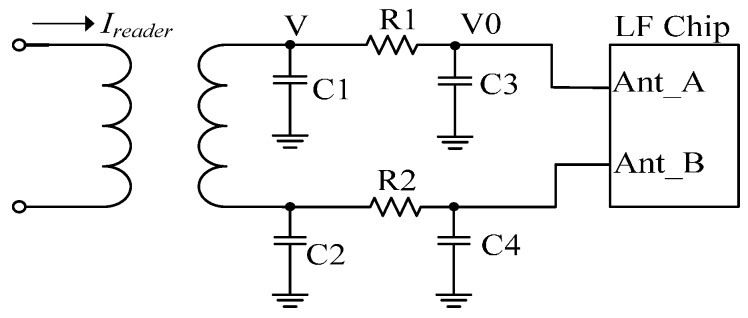
The front circuit of the low frequency chip.

The frontend circuit of the LF chip is one of the key components in low frequency communication. Before entering the RFID SIM LF chip, the signals need to be processed in a low-pass filter circuit, which is presented in Figure 5. The capacitors C1, C2, C3, C4, and the resistors R1 and R2 compose a filter circuit for filtering high-frequency noises. The values of the capacitors and the resistors need to be calculated and chosen discreetly when the system is in PWD and PDM mode. Besides the low frequency electromagnetic induction described by Equation (3), the resonance effect of the carrier is the key consideration. The resonance circuit consists of low frequency antenna, and capacitors C1 and C2. The resonant frequency is described by

(4) f=12πLC

where *f* denotes a resonant frequency equal to the carrier frequency, *L* is the inductance of the low frequency antenna in the SIM card, and *C* is the resonant capacitance including C1, C2, load capacitance, and parasitic capacitances. The inductance of the low frequency antenna of the SIM card is about 5 to 10 nH, and the capacitance of C1 and C2 can be calculated according to Equation (4).

The low-pass filter circuit consists of the resistors R1 and R2, and capacitors C3 and C4. The resistance of R1 and R2 cannot be too large in the low-pass filter circuit, which is described as

(5) V0=VjwR1(C3+CL)+1

where V0 denotes the voltage signal imported into the low frequency chip, V is the induced voltage after the antenna matching circuit, and *C_L_* means the loading capacitance of the low frequency chip. After many experiments, the optimized resistance of R1 and R2 is 30 to 50 ohm; this has a less negative impact on the weak induced voltage.

In our experiments, the working distance of low frequency communication is affected distinctly by the values of the resistors and capacitors in PDM and PWM modes. Nevertheless, this situation is not observed in the baseband transmission mode. Based on the foregoing analysis of the front circuit, this implies that resonance and load modulation effects should also be considered in PDM and PWM modes. After a long time conducting low temperature tests for approximately 72 h at −40 °C, we found that the transmission distance decreases by 1.5~2.5 cm in PDM and PWM modes. However, the communication distance for the same SIM card decreases by 0.5~1 cm in the baseband transmission mode. Therefore, the baseband transmission mode is a better scheme for the 2 kHz LF communication in RFID SIM card applications.

### 4.2. Programmable Gain Amplifiers of the LF Chip

**Figure 6:**
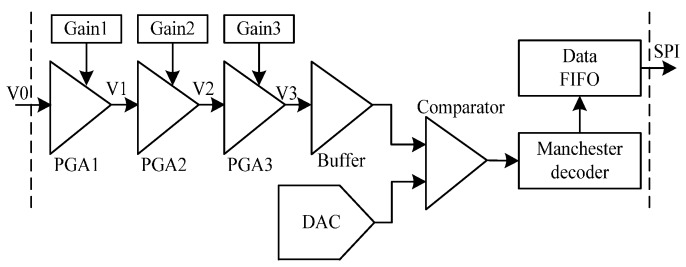
The structure diagram of the low frequency (LF) chip.

The LF signal process is described in Figure 6. The weak signal (V0) from the front circuit of the low frequency chip needs to be amplified from tens of micro volts to a few volts. The three-stage programmable gain amplifiers (PGA) and one buffer are applied to deal with it. The amplified voltage from the buffer and the output signal from the DAC are input into a comparator, which contributes to the high and low level decision of the inductive signal. The circuit is also used to determine whether a card approaches or departs, which in turn puts the 2.45 GHz chip in a waking up or sleeping mode accordingly. Thus, the bit stream produced by the comparator is decoded by the Manchester decoder [24]. The decoded data is checked by cyclic redundancy check (CRC) algorithm. The 8-bit CRC circuit is designed with the polynomial x^8^ + x^2^ + x + 1. Lastly, an interrupt signal from the LF chip is generated and sent to the SE chip, and the SE chip accesses the LF chip and fetches data by the serial peripheral interface.

**Figure 7:**
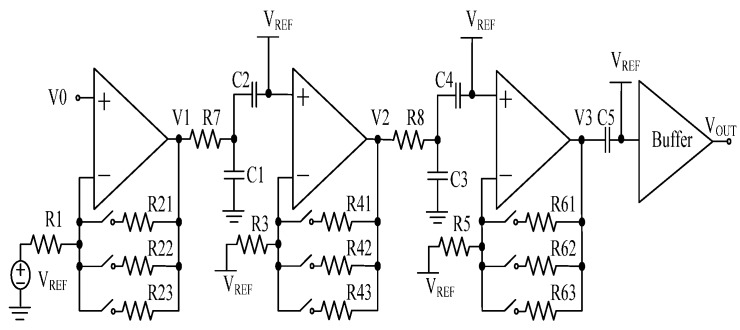
Programmable gain amplifiers circuit.

The PGA and buffer circuits are described in Figure 7. Each PGA circuit uses a resistive feedback network. For example, the first stage resistor network includes R1, R21, R22, and R23. The magnification is determined by the ratio of the feedback resistor to the gain resistor. The voltage gain can be controlled by the configuration registers, which choose different feedback resistors such as R21, R22, and R23. The second and third PGA has the same circuitry. Between each stage of PGAs, the low-pass filter circuit is carefully designed for removing high frequency noises, which contain R7, R8, C1, and C3. The coupling capacitors C2, C4, and C5 are applied to block the direct current level signal. The signal V3 is inputted into the rail-to-rail output buffer, which enhances the load capacity of the PGA circuit. There are two voltage band-gap (BG) references in the LF chip. One of them is connected to the input ends of the antenna, the PGAs, and the buffer as 1 V common-mode voltage in Figure 7. The 1.2 V BG works as the reference voltage, which is used to generate the digital and analog voltage sources.

**Figure 8:**
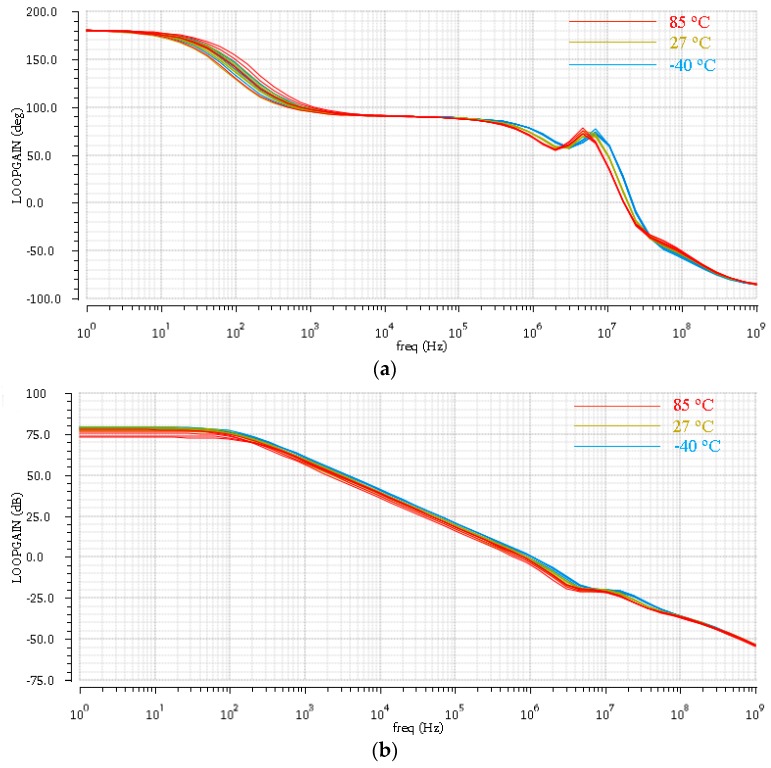
(**a**) Loop gain phase frequency response of programmable gain amplifiers (PGAs) at the corners of −40 °C, 27 °C, and 85 °C; (**b**) Loop gain frequency response of PGAs at the corners of −40 °C, 27 °C, and 85 °C.

Figure 8 describes the loop gain phase and the loop gain frequency response of the PGAs. We simulated the PGA circuits at −40 °C, 27 °C, and 85 °C for different process corners including slow, fast, typical, slow N fast P, and fast N slow P models. Therefore, all combinations result in 15 curves in Figure 8a,b. These curves show that the proposed PGA circuits work correctly in different temperatures and process corners, and achieve the requirement of the small signal amplification at 2 kHz.

### 4.3. Differential Manchester Decoder

A Differential Manchester code is a line code in which data and clock signals are combined to form a single 2-level self-synchronizing data stream. In the Differential Manchester code stream, 0 is represented by no transition and 1 is represented by a transition [25,26]. The Manchester decoder needs to estimate the long and the short signal level to judge the transition. The common method of Manchester decoding is that the signal level between two transitions is sampled and recorded by the sampling clock. Then, the decoder compares the number of samples and the threshold value of the signal length to distinguish between the long and short signal levels. Lastly, the symbols 0 and 1 are exported by a decoder. Accordingly, the sampling clock precision is very important to decode the code stream correctly, and some anti-noise methods are proposed [27,28]. A serious problem is that the clocks generated by the linear oscillator circuit used a resistive and capacitive network of the different LF chips are unstable and that different chips have different frequencies. In the worst case, the clock frequency deviation reaches from −20% to +20%, and this results in a higher Manchester decoding error rate.

**Figure 9:**
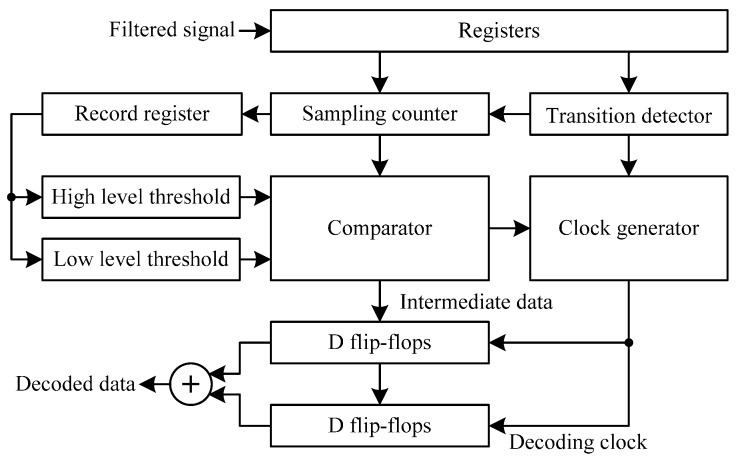
The architecture of the differential Manchester decoder.

**Figure 10:**
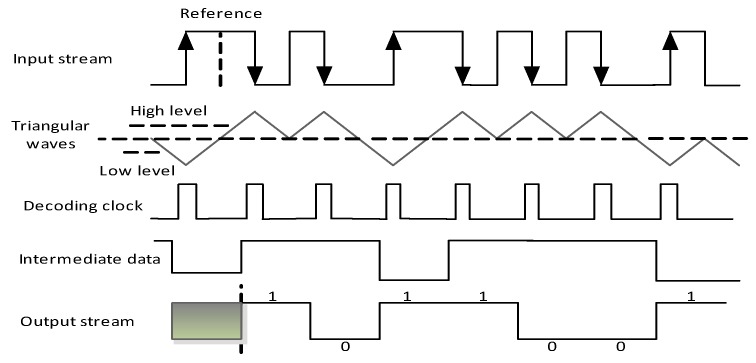
The Differential Manchester decoding process.

The proposed differential Manchester decoding method is illustrated in Figure 9. The decoding signal is first filtered by the filtering circuit to remove the glitches. In the sample counter circuit, when catching high level signals, the counter increases by 1; when sampling the low level signal, the counter decreases by 1. Consequently, the square waves are transformed into triangular waves, as illustrated in Figure 10. The high and low level threshold values are imported into a comparator, which is similar to the function of the Schmitt trigger. When the triangular waves are greater than the high threshold, the intermediate data is 1; when the triangular waves are less than the low threshold, the intermediate data is 0. The transition detector is used to generate the decoding clock. There are two conditions to generate a decoding clock. One is that the positive edge trigger occurs and the triangular waves are less than the low level threshold, the other is that the negative edge trigger occurs and the triangular waves are greater than the high level threshold. Then, the decoding clock is fed into the two groups of D flip-flops, which perform the two stages of the intermediated data sampling. Lastly, the decoded data is generated by the XOR operation of the two stages of the D flip-flops.

Three-fourths of the maximum value from the sampling counter is used as the high level threshold, and a quarter of that is applied as the low level threshold. When catching negative edges, the value of the sampling counter is imported to record the register. The SE chip reads values many times and calculates the mean value as the maximum value. Therefore, the SE chip sets the default value as the maximum according to the original clock frequency, and the value of the high and low level thresholds can be obtained adaptively according to the real clock frequency. When adopting triangular waves, the long and short signal levels are not divided by the fixed threshold values. The clock skew only causes a difference in the height of the wave. After using the adaptive high and low level threshold scheme, the effects caused by the clock frequency deviation are eliminated.

We added some test pins for testing the Manchester decoder. The test pin between the comparator and the Manchester decoder in Figure 6 is added and connected to a field programmable logic arrays (FPGA) board. The digital logic chips including the Manchester decoder, the data fist in fist out (FIFO) modules, the CRC circuit, and the SPI circuit are implemented by the FPGA. The expected digital clock frequency is 380 kHz, and our test frequency is from 270 kHz to 490 kHz at 1 kHz intervals. The clock is generated and controlled by a digital function generator, which is connected to the FPGA as the decoding clock. The experiment result shows that this circuit can decode successfully from 270 kHz to 490 kHz, which means this differential Manchester decoder can work under a clock skew of approximately ±30%.

## 5. Implementation and Experimental Results

**Figure 11:**
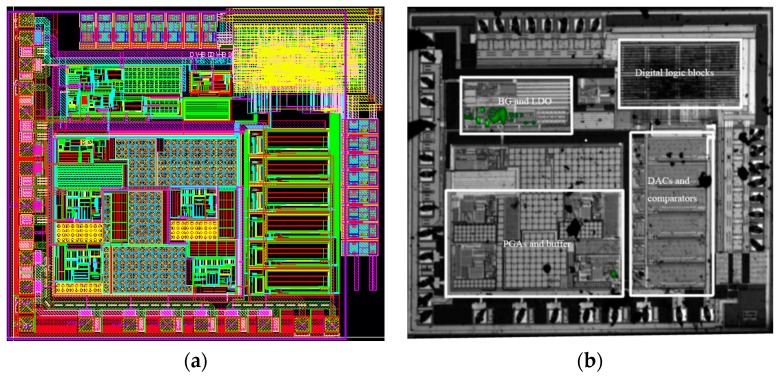
The layout and photograph of the low frequency chip (**a**) layout; (**b**) photograph.

The LF chip has been fully implemented in Central Semiconductor Manufacturing Corporation (CSMC) 0.18 µm mixed signal technology with one layer of polycrystalline silicon and five layers of metal. The die size is 1850 µm by 1650 µm with a 330 µA working current and a 45 µA idle current, which can satisfy the current requirements of the SIM card. Figure 11 gives the low frequency chip layout and photograph. The low frequency chip includes six DACs and comparators for different functions, such as the Manchester code stream generation, and approach and departure detection. Figure 11b also shows that there are some current hot spots near the band-gap and LDO chips in the low frequency chip.

**Figure 12:**
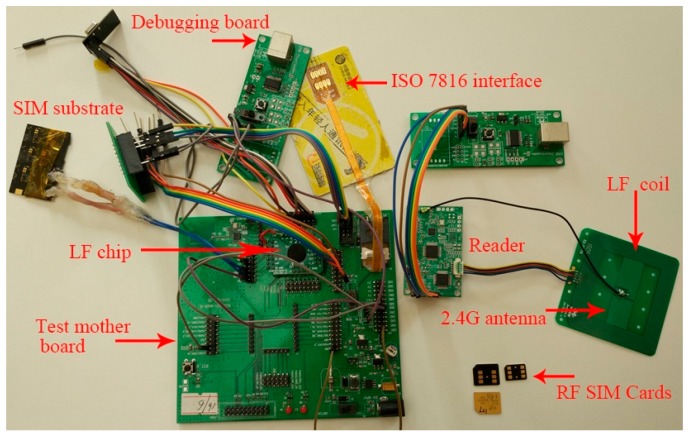
The low frequency chip test board of the RFID SIM cards.

Figure 12 shows the test platform of the LF chip and 2.45 GHz chips. To guarantee testing availability and reliability, the LF chip uses the coils from the real RFID SIM card substrate, and the reader is from the commercial products. The LF chip with the chip on board (COB) package is fitted into the test motherboard. The SIM substrate in the upper left corner consists of low frequency coils and the frontend circuit, which is connected to the LF chip and the motherboard via wires. The ISO 7816 interface is in the top right corner of the motherboard, and transfers data from the debug host. The debugging board is at the top, which provides power and the debugging interface for the motherboard. The dual-mode reader is on the right-hand side. The same debugging board is connected to the reader. To the far right is the antenna board for the reader, which contains low frequency loop coils and a 2.45 GHz high frequency rod antenna.

**Figure 13:**
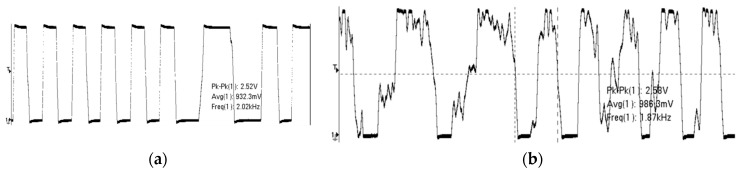
(**a**) Waveform of the normal decoding region; (**b**) Waveform of the critical decoding region.

We tested the communication distance and waveforms of the V3 signal in Figure 7 for the LF chip using the test platform. Figure 13 illustrates the waveforms of V3 for different distances between the reader antenna and the LF coil in the SIM card substrate. The waveform in Figure 13a is consistent with *V_SIM_* in Figure 3 described by the low frequency transmission model. Figure 13b shows that the wave distortion of V3 causes incorrect Manchester decoding in the critical region. In our tested experiments, the mean error rates are measured by the CRC error interrupt. The mean error rate is lower than 2% when the LF chip is in the normal decoding region. However, the mean error rate grows quickly beyond the critical decoding region, and decoding failures will cause the whole application to fail. Besides this, our test results also demonstrate that the chip has a better communication distance and decoding stability when the PGAs entering into the saturated amplification mode.

**Figure 14:**
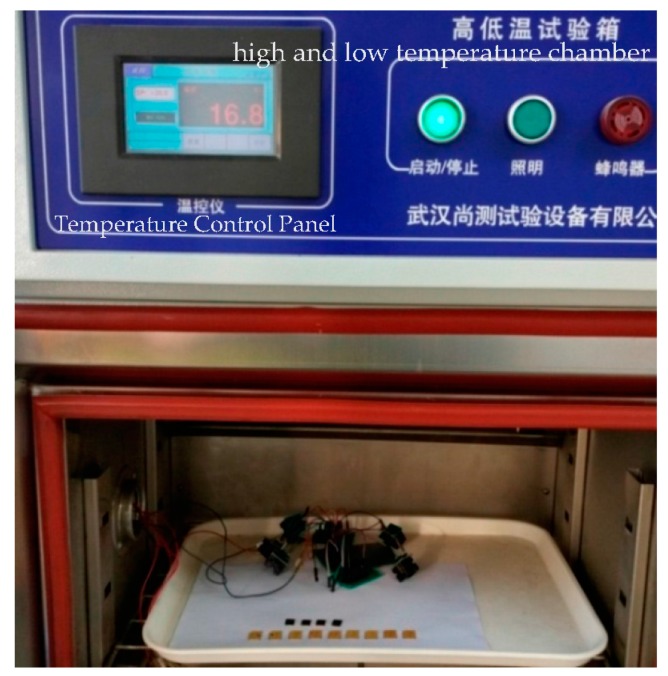
Temperature test chamber for RFID SIM cards.

The micro and nano-SIM cards are in the lower right corner of Figure 12, which show the same shape as the ordinary SIM cards. To integrate the secure element chip and low frequency chip, the back thinned process has been done for the low frequency circuit wafer and the SE wafer for the purpose of stacking. The RFID SIM cards can fit almost all mobile phones. To ensure the adaptability of working conditions, we performed temperature tests with −40 °C, −20 °C, 0 °C, 27 °C, 60 °C, and 85 °C for the RFID SIM cards. The temperature test platform is shown in Figure 14 including a high and low temperature chamber. We chose different groups of RFID SIM cards and placed them in the chamber for 72 h at each temperature. Then the different groups of RFID SIM cards are tested for communication distances respectively in the air, and by using the test board of the RFID SIM cards. The test results are illustrated in Table 2, which shows the working distances in the baseband transmission, PWM, and PDM modes for different temperatures. The experiment reveals that the baseband transmission is a better working distance and more excellent temperature adaptability, and that high temperatures have less influence on the working distances of RFID SIM cards than low temperatures. The RFID SIM cards in the BBT mode show good performance at almost all the temperatures, but there exists a small decrease for communication distances at −40 °C.

To study the effects of low temperature for the LF communication, we used 10 COB LF chips and SIM substrates with antennas to do some experiments. Firstly, the electrical parameters of the LF chips are tested at 27 °C, and depicted in Table 3. The parameters are measured from the testing pins of COB chips, including digital device voltages (D_VDD), analog device voltages (A_VDD), voltage source references from BG (V_BG), PGAs’ common-mode voltages from BG (P_BG), the frequencies of internal oscillators, and the working current of the LF chips. Then, the chips and substrates are placed into a temperature chamber for 72 h at −40 °C. The LF chips are tested again and the results are shown in Table 4. Apart from the working current, all the variations of the electrical parameters are no more than 0.3% from Table 3 and Table 4. The mean working current increased by 2.7% and communication decreased by 6.7% for BBT mode, which shows there is a possible correlation between the variation of the working current and that of the distance. Besides this, when we changed a new SIM substrate with the front circuit and LF coils without staying at the low temperature, the working distance showed some improvements for PWM and PDM modes, but very little change for BBT modes.

Therefore, the low temperature at −40 °C will bring about many adverse influences on the LF transmission, first of which is the values of capacitors and resistors in the front circuit change, which causes a decrease in the working distance in PDM and PWM modes. Secondly, the variations of the working current imply a leakage current increase of the LF chips. Lastly, some changes of the circuit parameters in the LF chip and increased noise by leakage current possibly affect the amplification quality for the weak signal. All these lead to working distance degradation at low temperature after a long period.

We installed the dual-mode reader into the retail POS (point of sale) terminal of the Newcapec Company, and compared the communication distance and success ratio of Manchester decoding for the RFID SIM card at room temperature in air and in different mobile phones, including Apple, Huawei, and Samsung. The test results are summarized in Table 5, which shows that the LF chips have excellent near field communication ability and can work within a 7 cm range for almost all popular mobile phones. The NFC works at distance about 6 cm for the Apple iPhone 6S with the Newcapec POS terminal. SIM cards in air have the longer working distance and the higher decoding success ratio. The metal shell shielding of mobile phones has more influence on the working distance and the decoding success ratio than plastic shells. The experiments also show that if the decoding success ratio is more than 80%, the payment application can work correctly because the LF reader sends the same waking up and configuration data repeatedly.

## 6. Conclusions

The integration of the 2.45 GHz RFID chip and the LF chip for RFID SIM cards suitable for middle and long distance RFID applications and near field payments has been discussed and implemented. Compared to ordinary SIM and e-SIM cards, RFID SIM cards consist of a secure element chip, a 2.45 GHz RF chip, and a low power range controlled communication chip with a 2 kHz working frequency. This RFID SIM can support both long distance and near distance applications with reasonable power consumption. For future work, there are some issues worthy of further study in the LF chip design, for example the mechanism for working distance degradation at low temperature; the metal shielding effect of mobile phone shells; the relationship between the working distance and the direction of operation; the reduction of communication crosstalk between a low frequency and 13.56 MHz; and the trade-off between power consumption and working distance at different operating frequencies (e.g., 4 kHz, 8 kHz, and 10 kHz).

## Figures and Tables

**Table 1:** Working distances of RFID SIM for three different modes in air.

Distance (cm)	BBT	PWM	PDM
Micro-SIM	13.5	9.2	12.7
Nano-SIM	12.0	8.0	11.4

**Table 2:** Working distances of nano-SIM cards at different temperatures in air for the baseband mode.

	−40 °C	−20 °C	0 °C	27 °C	60 °C	85 °C
Distance in BBT (cm)	11.2	11.8	12.0	12.0	12.0	12.0
Distance in PWM (cm)	6.5	7.9	7.9	8.0	7.9	7.9
Distance in PDM (cm)	9.2	11.2	11.3	11.4	11.3	11.3

**Table 3:** Electrical parameters of the LF chips at 27 °C.

No.	D_VDD (V)	A_VDD (V)	V_BG (V)	P_BG (V)	OSC (kHz)	Current (µA)
1	1.799	1.960	1.150	0.989	402.17	325.5
2	1.844	1.996	1.165	0.974	391.02	329.1
3	1.785	1.954	1.144	0.991	391.74	328.5
4	1.838	2.000	1.167	1.007	402.4	335.2
5	1.850	1.975	1.169	1.019	376.94	333.4
6	1.801	1.943	1.134	1.020	372.33	327.3
7	1.857	2.000	1.173	1.009	388.56	229.7
8	1.819	1.967	1.161	1.008	376.56	324.3
9	1.856	2.000	1.171	0.982	355.17	341.2
10	1.824	1.969	1.159	0.989	382.43	325.5

**Table 4:** Electrical parameters of the LF chips after 72 h at −40 °C.

No.	D_VDD (V)	A_VDD (V)	V_BG (V)	P_BG (V)	OSC (kHz)	Current (µA)
1	1.801	1.963	1.151	0.99	401.95	331.5
2	1.848	1.994	1.167	0.974	391.3	340
3	1.782	1.955	1.147	0.993	391.53	330.4
4	1.846	2	1.184	1.007	402.67	339.4
5	1.854	1.977	1.172	1.02	377.61	338.8
6	1.805	1.947	1.14	1.024	372.35	331
7	1.864	2	1.176	1.01	386.24	335.8
8	1.824	1.966	1.163	1.011	376.55	328.5
9	1.859	2	1.172	0.982	356.58	347.8
10	1.820	1.963	1.155	0.986	381.56	330.4

**Table 5:** Working distances and Manchester decode success ratio of RFID SIM for different mobile phones and in air.

	Iphone 6	Iphone 5s	Huawei Mate 7	Samsung I9200	In Air
Distance (cm)	7.8	8.2	7.5	8.8	12
Success ratio	93%	95%	91%	98%	99%

## References

[1] Want R. (2010). An Introduction to RFID Technology. IEEE Pervasive Comput..

[2] Tan W.H., Ooi K.B., Chong S.C., Hew T.S. (2014). NFC mobile credit card: the next frontier of mobile payment?. Telemat. Inform..

[3] Pasquet M., Reynaud J., Rosenberger C. Secure Payment with NFC Mobile Phone in the Smart Touch Project. Proceedings of the International Symposium on Collaborative Technologies and Systems.

[4] Bolhuis M. Using an NFC-Equipped Mobile Phone as a Token in Physical Access Control. http://essay.utwente.nl/65419/1/thesis_nfc_martijn_bolhuis_final.pdf.

[5] Liu F., Janssens D., Wets G., Cools M. (2013). Annotating mobile phone location data with activity purposes using machine learning algorithms. Expert Syst. Appl..

[6] Henpraserttae A., Thiemjarus S., Marukatat S. Accurate Activity Recognition Using a Mobile Phone Regardless of Device Orientation and Location. Proceedings of the 2011 International Conference on Body Sensor Networks.

[7] Yang G., Huang Z., Wan L. The development of RFID module in NFC phone. Proceedings of the International Conference on Anti-Counterfeiting, Security, and Identification in Communication.

[8] Von Oheimb D., Lotz V., Walter G. (2005). Analyzing SLE 88 memory management security using Interacting State Machines. Int. J. Inf. Secur..

[9] Hsu C.C., Chen J.H. (2011). A novel sensor-assisted rfid-based indoor tracking system for the elderly living alone. Sensors.

[10] Kim S.H., Park H., Bang H.C., Kim D.H. (2014). An indoor location tracking based on mobile rfid for smart exhibition service. J. Comput. Virol. Hacking Tech..

[11] Ni L.M., Liu Y., Lau Y.C., Patil A.P. LANDMARC: Indoor location sensing using active RFID. Proceedings of the IEEE International Conference on Pervasive Computing and Communications.

[12] Qing X., Chen Z.N. (2007). Proximity Effects of Metallic Environments on High Frequency RFID Reader Antenna: Study and Applications. IEEE Trans. Antennas Propag..

[13] Lee B., Kim B., Mum B., Lee H. (2014). NFC Antenna Design for Low-permeability Ferromagnetic Material. IEEE Antennas Wirel. Propag. Lett..

[14] Yu S., Peng Y., Huang X. Realization of Coordinative Control between Multi Readers and Multi RF-SIM Cards under Mobile RF-SIM Mode. Proceedings of the International Conference on Computer Science and its Applications.

[15] Ying L.I., Hong L.I., Chen Y.T. (2014). Design on highway charge system based on RFSIM path recognition. Mod. Electron. Tech..

[16] Dai W., Luo G., Liu S. A new technology of short-range communication for one card solutionin school. Proceedings of the IET International Conference on Communication Technology and Application.

[17] Wei Q., Wang X. The Design and Implementation of the Minimal M2M Terminal Based on the SIM4100 Wireless Module. Proceedings of the Third International Symposium on Information Processing.

[18] Shan L., Li Z., Hu H. (2012). Converged Mobile Cellular Networks and Wireless Sensor Networks for Machine-to-Machine Communications. Ksii Trans. Internet Inf. Syst..

[19] Smart Cards Embedded UICC Requirements Specification (Release 13): ETSI TS 103.383. http://www.etsi.org/index.php/technologies-clusters/technologies/smart-cards.

[20] Remote Provisioning Architecture for Embedded UICC Technical Specification Version 3.1: GSMA. SGP.02. http://www.gsma.com/newsroom/all-documents/sgp-02-v3-1-remote-provisioning-architecture-for-embedded-uicc-technical-specification/.

[21] RSP Technical Specification Version 1.1: GSMA. SGP.2. http://www.gsma.com/newsroom/all-documents/sgp-22-technical-specification-v1-1/.

[22] Understanding SIM Evolution: GSMA. https://www.gsmaintelligence.com/research/2015/03/understanding-sim-evolution/499/.

[23] Klaus F., Müller D. (2010). RFID Handbook: Fundamentals and Applications in Contactless Smart Cards, Radio Frequency Identification and Near-Field Communication.

[24] Chen Y., Zheng Z. A low-power 2K/4K range-controlled communication chip design for mobile payment. Proceedings of the IEEE International Nanoelectronics Conference.

[25] Hung Y.C. (2012). Time-interleaved CMOS chip design of Manchester and Miller encoder for RFID application. Analog. Integr. Circuits Signal Process..

[26] Saltzberg B. An improved Manchester code receiver. Proceedings of the IEEE International Conference on Communications.

[27] Guo X., Chen Z. (2014). A New Circuit for Manchester Decoder Based on FPGA. Open J. Circuits Syst..

[28] Xu D.C., Lu J.F. (2012). Design of Manchester codec based on FPGA. Adv. Mater. Res..

